# Driving Forces of UCST and LCST Liquid–Liquid Phase Separations

**DOI:** 10.1021/acsomega.6c05528

**Published:** 2026-08-10

**Authors:** Hussen O. Mohammed, Ronen Zangi

**Affiliations:** † 16402 Donostia International Physics Center (DIPC), Donostia-San Sebastián 20018, Spain; ‡ Department of Organic Chemistry I, University of the Basque Country UPV/EHU, Donostia-San Sebastián 20018, Spain; § IKERBASQUE, Basque Foundation for Science, Bilbao 48009, Spain

## Abstract

A liquid mixture of two or more components can phase separate into two liquid phases by changing the temperature. Ordinary chemical perception would attribute this transition to a competition between the favorable enthalpy of the biphasic state and the favorable entropy of the (mixed) monophasic state, rationalizing the stability of these states, respectively, at low and high temperatures. This is indeed the case for a UCST liquid–liquid phase separation. However, in contrast, in LCST behavior, the monophasic mixed state is observed at low temperatures, whereas the biphasic state is observed at high temperatures. This perplexing behavior has triggered different hypotheses about mechanisms capable of explaining it. In this paper, we study, by molecular dynamics simulations, two ionic liquids (ILs), [Hbet]­[Tf_2_N] and [P_4444_]­[TMBS], that when mixed with water exhibit, respectively, a UCST and an LCST liquid–liquid phase separation. The computational phase diagrams of these two IL/water mixtures were shown to be in very close agreement with those obtained experimentally. Here, we investigate the effective interactions between the ions in aqueous solutions at a very large dilution. We find that for an LCST IL, an increase in temperature induces substantial strengthening of the effective cation–anion and cation–cation attractions (whereas the anion–anion interactions are not significantly altered). Conversely, a UCST IL displays marginal changes in the effective interactions between the ions with a change in temperature. Calculations of thermodynamic parameters indicate that for an LCST IL, the driving force for forming cation–anion contact pairs is predominantly entropic. This positive entropy change for association, which we also attribute to the formation of the biphasic state, does not include the mixing entropy and is ascribed to changes in the properties of surrounding water molecules. On the other hand, for a UCST IL, the driving force for cation–anion association is purely enthalpic. These findings are supplemented by demonstrating that the reorientations and translations of water molecules in the first solvation shell around the LCST ions (in particular, around the cation) are slower than those around the corresponding UCST ions. Given the anomalous behavior of strengthening effective interactions upon heating, we conclude that the transition of a mixed homogeneous state to a liquid–liquid phase-separated state upon a temperature increase (LCST behavior) is driven primarily by hydrophobic interactions.

## Graphical abstract

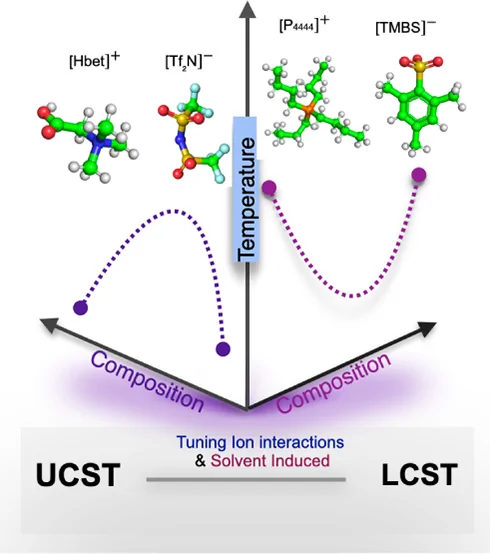

## Introduction

Segregation of a mixture into two liquid phases is a phenomenon known to chemists for a very long time. Recently, though, this field has experienced a renaissance due to discoveries indicating the formation of membraneless compartments inside biological cells^1^ and due to developments of thermoresponsive materials (such as ionic liquids) that, when mixed with solvent (water), phase separate only within a (potentially tunable) range of temperatures.^2^

When thinking about the factors driving liquid–liquid phase separation, the loss of mixing entropy naturally arises. This mixing entropy favors the formation of a mixed monophasic state and opposes the formation of a biphasic state. Yet, phase separation can spontaneously form if the enthalpy gain in transforming to the biphasic state compensates for this entropic penalty of demixing. Because the contribution of entropy to the Gibbs free energy becomes more important at higher temperatures, it is expected that if phase separation is possible, it will be observed at low temperatures, whereas the single mixed phase is at high temperatures. This is indeed the behavior of UCST (upper critical solution temperature) liquid–liquid phase separation.^3–8^ However, counterintuitively, in LCST (lower critical solution temperature) behavior, phase separation is induced by *raising* the temperature,^9,10^ a phenomenon that, throughout the years, has intrigued many scientists and stimulated them to suggest physical mechanisms capable of explaining it.

An early and often-cited explanation proposed that in systems displaying LCST behavior, the two distinct chemical components in the mixture interact with each other via strong directional forces, such as hydrogen bonds.^11^ With an increase in temperature, these short-range hydrogen bonds between unlike molecules are easily broken. Nonetheless, if one of the separated phases can better retain its hydrogen bonding with an increase in temperature, the overall enthalpy of the biphasic state is more favorable than that of the mixed phase, and hence, segregation can be driven by a gain in enthalpy.^12,13^ For example, in simulations of [P_4444_]­[CH_3_COO]/water mixtures, it is claimed that the hydrogen bonds between the anion and water were drastically destroyed with increasing temperature, leading to aggregation of cations and anions.^14^ A similar mechanism is stated for the LCST behavior of ternary mixtures consisting of two different amino acid ILs and water, where anion-water interactions are said to be weakened, whereas anion–anion interactions between the different amino acids are strengthened with increasing temperature.^15^ A hydrogen bond that breaks at higher temperatures is also stated to trigger the formation of a biphasic state of poly­(ethylene oxide) (PEO) in the ionic liquid [BMIM]­[BF_4_].^16^ Nevertheless, here, the weakened PEO-cation hydrogen bond is argued to be a result of an entropic penalty.

An alternative explanation for the puzzling LCST behavior suggests that the monophasic phase is not purely random (or homogeneous) but composed of small ordered molecular clusters formed by one of the components in the system.^17–19^ This nanoscale organization obviously reduces the entropy of the “mixed” state, and in the absence of similar ordering in the segregated phases, the (temperature-induced) transformation to a biphasic state can be driven by a gain in entropy. In a somewhat related explanation, it is argued that LCST behavior observed in water mixtures is also induced by an increase in entropy; however, the increase in entropy here is due to ordering of water molecules around hydrophobic residues of the second component, rendering the entropy of the biphasic phase larger.^20^ On a similar note, by observing both types of phase separation in polyelectrolyte complexes, it is claimed that a temperature increase in UCST behavior transforms the system into the mixed state by the dissociation of cation–anion attractions.^21^ In contrast, a temperature increase in LCST behavior transforms the system to the biphasic state as a result of dehydration-induced association of cation and anion.^21^ Another study found that the excess Gibbs energy of the solvent exhibited contrasting contributions in LCST and UCST regimes.^22^ It is interesting to point out that in polymer/ionic-liquid and ion-gel systems, exhibiting liquid–liquid phase separations, the driving forces for the transitions have also been explained in terms of the temperature dependence of the Flory–Huggins interaction parameter χ, as well as by using concepts of free-volume theory.^23^ Other mechanisms aiming to explain LCST phase separations are reported in the literature,^24–28^ as well as a study focused on elucidating the phase separation kinetics of this transition.^29^

All-atom explicit-solvent molecular dynamics simulations were applied to investigate the behavior of ionic liquids (ILs) that, when mixed with water, displayed temperature-induced liquid–liquid phase separation. For a mixture of [Hbet]­[Tf_2_N] (protonated trimethylglycine bis­(trifluoromethylsulfonyl)­amide) and water, the phase diagram obtained from the simulations is of the UCST type,^30^ whereas for a mixture of [P_4444_]­[TMBS] (tetrabutylphosphonium 2,4,6-trimethylbenzenesulfonate) and water, an LCST phase diagram is observed^31^ (see Figure 1 for the chemical structures of these ILs). Both of these thermoresponsive ILs have been shown experimentally to be potential draw solutes in the seawater forward-osmosis desalination process, where the critical temperatures are not far from room temperature.^32,33^ This potential application, the high appearance of these cations and anions in IL/water mixtures exhibiting UCST and LCST behavior,^34^ and the availability of detailed experimental phase diagrams led us to choose them as representative materials to study these two types of liquid–liquid phase behavior. For both of these UCST and LCST IL/water mixtures, the computed phase diagram agrees very well with that found experimentally.^2,32,35^ This is shown in Figure 2 where we replot these two types of phase diagrams. Note that the compositions of the IL-rich and water-rich phases were determined only by considering the regions of these phases, slightly different than the original analysis performed for [P_4444_]­[TMBS]/water, in which case the interface regions were also taken into account.^31^ Furthermore, to model a classical force-field charge transfer from anions to cations, which has been demonstrated to take place in neat IL phases,^36–40^ the unit charges of these ions were scaled down by a factor of 0.95. Within a narrow range of ±0.05 *e*, this was the only charge scaling value that could qualitatively support the monophasic to biphasic transformation. As it turns out, scaling the charges of the ions by this factor also yielded a *quantitative* agreement with experimental data, where all features of both phase diagrams were reproduced by the simulations, albeit with an overestimation of the critical temperature by 10 K for the case of the [P_4444_]­[TMBS]/water mixture (Figure 2).

**Figure 1:**
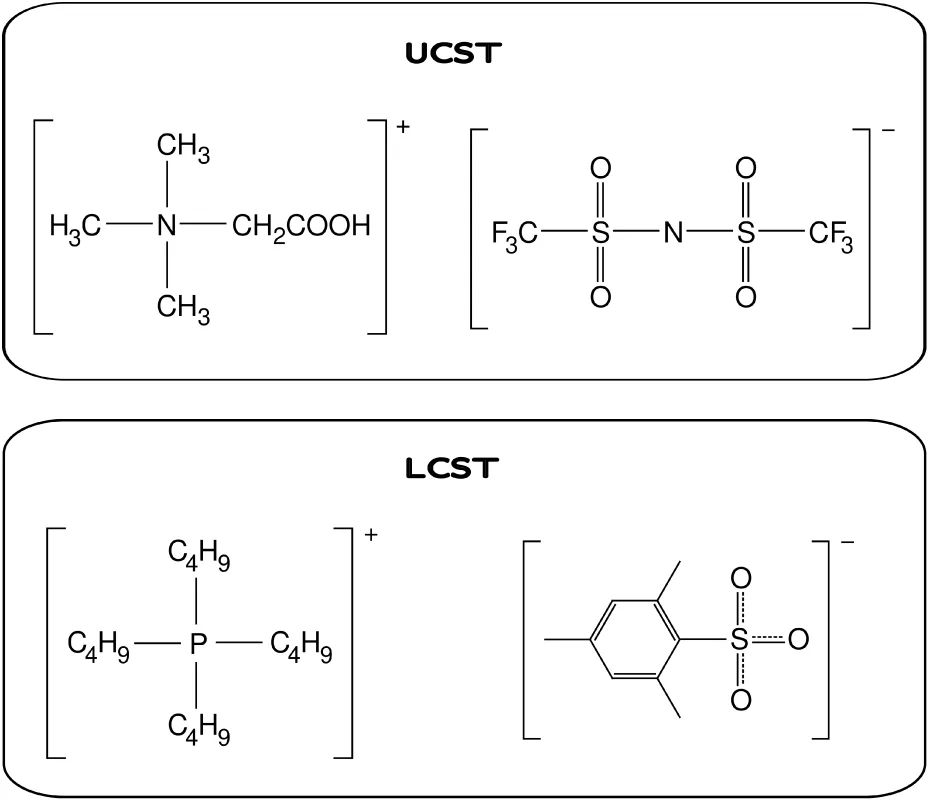
Chemical structures of [Hbet]­[Tf_2_N] (top panel) and [P_4444_]­[TMBS] (lower panel) ILs that, when mixed with water, exhibit, respectively, a UCST and an LCST liquid–liquid phase separation.

**Figure 2:**
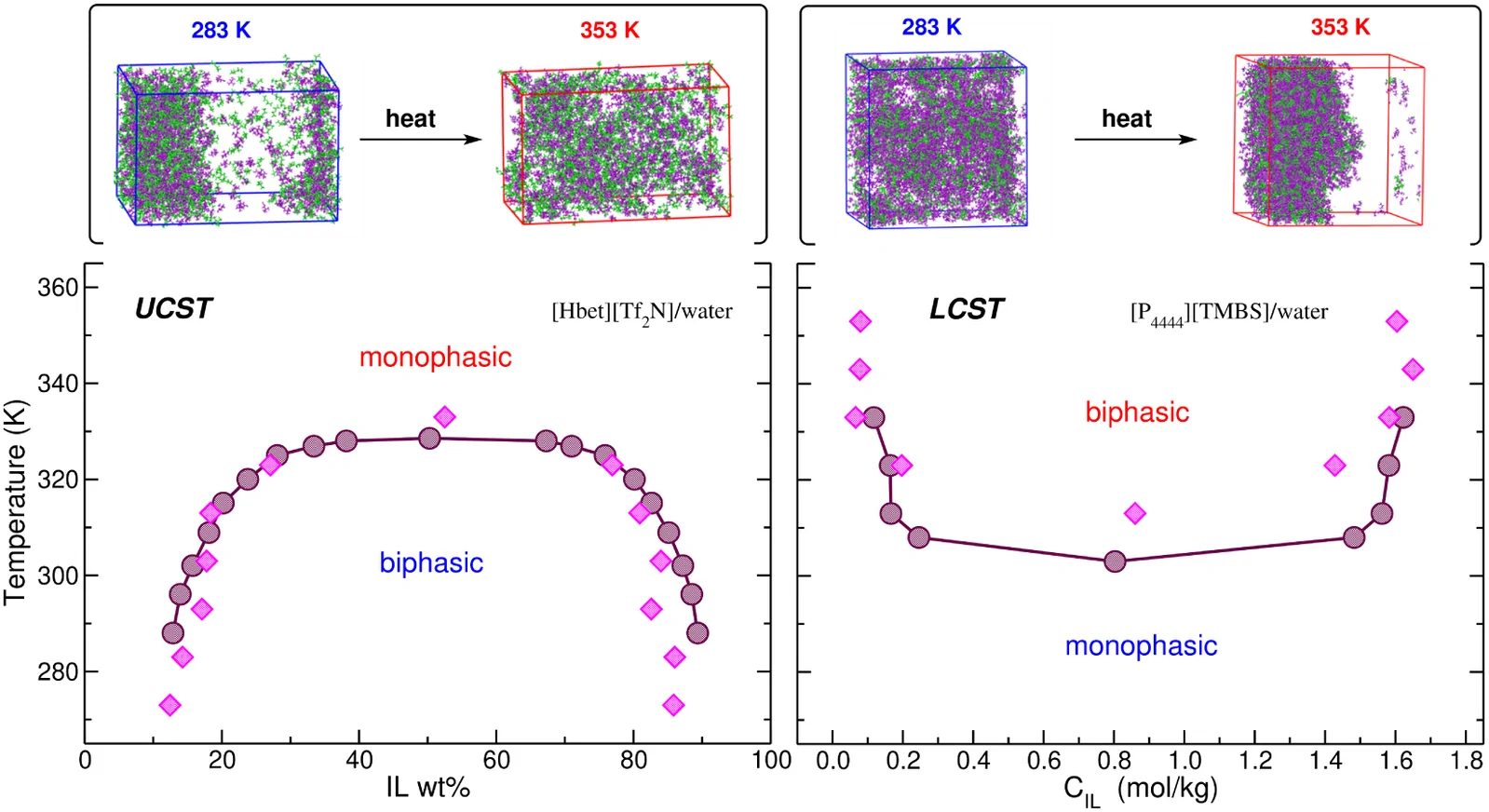
Lower panel: temperature–composition liquid–liquid phase separation diagrams of [Hbet]­[Tf_2_N]/water mixture displaying a UCST-type behavior (left) and of [P_4444_]­[TMBS]/water mixture displaying an LCST-type behavior (right). Maroon circles are experimental data points,^2,32,35^ whereas magenta diamonds are simulation results.^30,31^ The lines connecting the experimental data points are guides to the eye. Top panel: snapshots of the simulation box (water molecules are omitted for clarity) of the monophasic and biphasic states for the two IL/water mixtures.

## Results and Discussion

We now aim to identify the driving force that governs the monophasic to biphasic transformation in an LCST IL mixed with water and differentiate it from that leading to a UCST behavior. For this purpose, we look at the effective interactions between the respective ions in aqueous solution at large dilution. To facilitate the assessment of like-charge ionic interactions,^41^ we simulate 2 cation–anion pairs in a box containing 10,000 water molecules (corresponding to an IL concentration of 0.011 M) at two different temperatures, 283 K and 353 K. For both the UCST and LCST IL/water mixtures mentioned above (thus, when the IL concentrations are much higher), these two temperatures correspond to different states. The pair potential of mean force (PMF), *w*(*r*), is calculated from the radial distribution function, *g*(*r*) (shown in Figure S1 of the Supporting Information), via the relation *w*(*r*) = −*RT* ln *g*(*r*). See Supporting Information for Computational details.

The cation–anion, cation–cation, and anion–anion effective interactions are shown in Figure 3. For UCST behavior ([Hbet]­[Tf_2_N]/water mixtures), the cation–anion effective interactions at low and high temperatures are similar, exhibiting attraction at close approach (0.4–0.8 nm) with a potential well of approximately 4.5 kJ/mol deep. Yet, the global (second) minimum at 353 K is marginally deeper (∼0.5 kJ/mol) than that at 283 K. Expectedly, both of the like-charge effective interactions are everywhere repulsive, where the repulsion is slightly stronger at higher temperatures. Taken together, these observations indicate that for the UCST IL, phase separation at low temperatures is possible because of the relatively strong cation–anion attraction at contact. With an increase in temperature, this cation–anion attraction (or any other effective interaction) is not significantly altered; however, the growing importance of the mixing entropy can render the formation of a single mixed phase more stable. In contrast, in LCST behavior ([P_4444_]­[TMBS]/water mixture), there is a substantial strengthening of the cation–anion effective attraction with an increase in temperature. This is evident by a deeper (∼2.0 kJ/mol), as well as wider, minimum of the PMF that represents the contact pair. Even a stronger strengthening is observed for the cation–cation interactions when the temperature is increased from 283 K to 353 K. In this case, the minimum of the contact pair is approximately 3.5 kJ/mol deeper, and the range of the attraction is significantly wider. Note that because of the four butyl groups around the phosphorus atom, the minimum of the cation–cation contact pair is seen at a large distance of around 0.96 nm, and it does not include intervening water molecules. On the other hand, the anion–anion PMF at low and high temperatures is quite similar, with a marginally more stable contact pair at the higher temperature. The minimum of the anion–anion contact pair is also observed at a large distance (more specifically, at 283 K, in the range 0.8–1.0 nm); however, here, configurations corresponding to the higher-end distances do include intervening water molecules. Everything considered, an increase in temperature clearly strengthens the effective attractions between the LCST IL. These effective interactions include the entropy change due to the reorganization of the solvent molecules upon ion’s association; however, they do not include a mixing entropy. Therefore, if this gain in effective interactions overcompensates the entropic penalty of demixing, liquid–liquid phase separation at higher temperatures will spontaneously take place.

**Figure 3:**
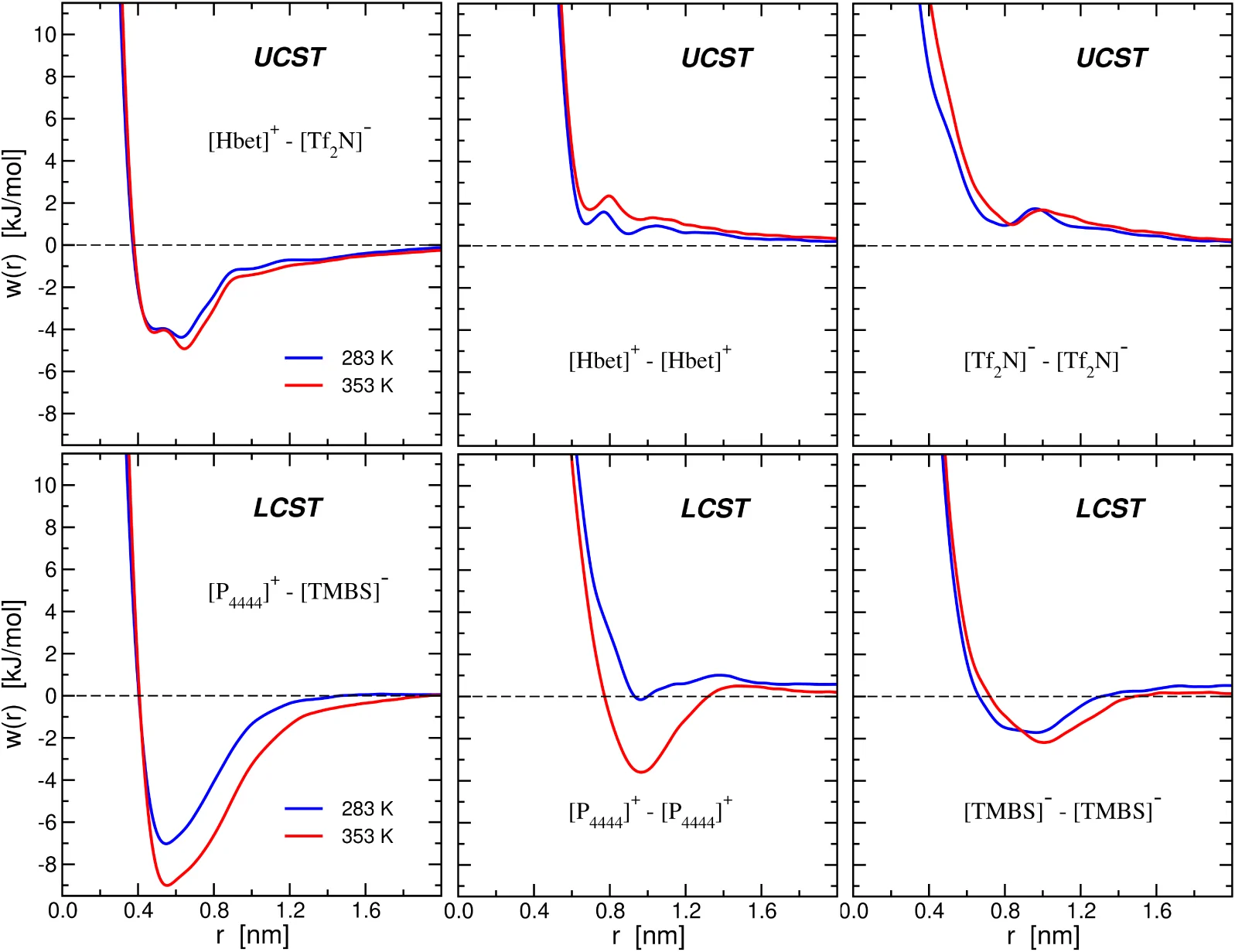
Potentials of mean force between the IL’s ions at low (283 K) and high (353 K) temperatures for UCST ([Hbet]­[Tf_2_N] mixed with water; upper panel) and LCST ([P_4444_]­[TMBS] mixed with water; lower panel) types of liquid–liquid phase separation. The results are obtained from a system containing only 2-IL units dissolved in 10,000 water molecules. In these calculations, the ions are represented by their “central” atoms: N for [Hbet]^+^, N for [Tf_2_N]^−^, P for [P_4444_]^+^, and S for [TMBS]^−^.

The fact that [P_4444_]^+^–[P_4444_]^+^ effective interactions displayed the largest strengthening of attraction with a temperature increase suggests the underlying driving force originates from hydrophobic interactions, in agreement with the conclusion made by Koga and coworkers studying *N*-isopropylpropionamide in aqueous solutions.^20^ The identification that hydrophobic interactions drive LCST behavior has also been proposed for the liquid–liquid phase separation observed for the amyloid tau protein at high salt concentrations.^42^ The existence of strong hydrophobic interactions between the cations is due to the four butyl groups on each ion. To a lesser extent, hydrophobic interactions are also present between [P_4444_]^+^ and [TMBS]^−^ (where the four butyl groups of the cation can interact with the trimethylphenyl group of the anion), and accordingly, the strengthening of the attraction is smaller. Hydrophobic residues are also present in the ions inducing the UCST behavior, [Hbet]^+^ (three methyl groups) and [Tf_2_N]^−^ (two trifluoromethyl groups). In fact, some degree of hydrophobicity of the IL seems to be a requisite to support liquid–liquid phase separation in general. However, in the case of the UCST IL, the contribution of the hydrophobic residues to the overall properties of the ions is smaller, and in consequence, no substantial strengthening in the effective attraction is detected upon an increase in temperature. Deguchi et al. discussed several aspects of the hydrophobicity/hydrophilicity balance of ILs and their relation to supporting LCST or UCST phase change.^2^

Instantaneous representative configurations of the interactions between the ions of these LCST and UCST ILs are shown in our previous studies.^30,31^ Although these configurations were extracted from mixtures with higher concentrations (that are able to phase-separate), the configurations we observed in the current study at large dilutions are similar. We note that, in the case of LCST ions, these instantaneous structures validate that substantial hydrophobic surface area is eliminated upon association. Qualitative visual inspections^31^ indicate that the size of the area eliminated decreases for the following series of ion pairs: cation–cation, cation–anion, and anion–anion. For UCST ions,^30^ it is the solvent-exposed surface areas of one (or more) of the methyl groups of the cation and/or the trifluoromethyl groups of the anion that are eliminated upon formation of the different ion pairs. It is interesting to note that at high temperatures the same binding modes as those found at low temperatures are operative, and therefore, it is predominantly the strength of interactions that are altered with a change in temperature. We also point out that when analyzing the radial distribution functions between the cation or anion and water (for the systems studied here at large dilutions, plots are not shown), both the UCST and LCST ions exhibited a decrease in the strength of the effective interactions. Although the peaks of the RDFs were observed at the same locations, at the higher temperature, their magnitudes were smaller.

It is worth emphasizing that these effective pair interactions between the ions are computed at very low concentrations, in which case phase separation is not possible, and therefore, their behavior as a function of temperature can serve as a predictor for phase behavior taking place at higher concentrations of the IL. Having said that, obviously not every aspect of the phase behavior can be addressed by the effective pair interactions at infinite dilution. For example, the PMFs of UCST and LCST ILs at 283 K indicate that the effective interactions between the LCST ions are at least as attractive as, if not more than, those between the UCST ions. Thus, if the latter displays phase segregation at this temperature, then it is expected that the former will phase separate as well. Note, however, the critical concentration (expressed by the IL/solvent molar ratio) is almost double for [Hbet]­[Tf_2_N], 1:20,^43^ compared with that for [P_4444_]­[TMBS], 1:39,^44^ pointing to the multidimensional character of the relative stability of the monophasic and biphasic states.

Two hydrophobic particles are effectively attracted to each other not because of direct interparticle attraction (which may or may not be present), but because the water molecules surrounding these particles experience reduced entropy relative to bulk water; in other words, the attraction is solvent-induced.^45–47^ In order to calculate the thermodynamics of the association process between a cation and an anion, we further simplified our system and included only 1-IL unit surrounded by 10,000 water molecules (corresponding to a concentration of 0.0055 M). The cation–anion PMFs (as well as the radial distribution functions) are shown in Figure S2 for UCST and LCST ILs. To define the changes of the thermodynamic parameters, we consider a process in which a cation and an anion approach one another from far apart (i.e., dissociated) to form a contact pair (associated). The value of Δ*G* of this reaction is calculated by subtracting the value of the PMF at a large distance (*r* = 3.0 nm) from the depth of the global minimum at contact (*r* = 0.65 and 0.55 nm for UCST and LCST ions, respectively). Owing to the negligible change in the volume of the system, the change in enthalpy, Δ*H*, is approximated by the change in energy. Next, the value of *T*Δ*S* is then derived from the relation Δ*G* = Δ*H* – *T*Δ*S*. The results are summarized in Table 1. As obtained from the 2-IL system, also here, the cation–anion contact pair exhibits stronger attraction at the higher temperature for both IL types, where the strengthening is significantly larger for LCST IL. Despite favorable formation of contact pair in both cases, the driving force for association is different for the different ILs. For UCST IL, the association is driven by enthalpy and opposed by entropy. For LCST IL, both enthalpy and entropy drive the association, with the latter being the dominant component at both temperatures. Note that, similar to the 2-IL systems, also here in the 1-IL systems, the changes in the thermodynamic quantities do not correspond to the process of liquid–liquid phase separation but to the association of a cation with an anion in a highly dilute aqueous solution. In particular, the value of Δ*S* extracted from this single ion-pair association process does not include the mixing entropy encountered when a biphasic state transforms to a monophasic mixed state. That means the entropy change of the monophasic to biphasic transformation of a many IL system, observed for LCST behavior upon heating, would be roughly additive over that obtained from a single ion-pair formation minus the mixing entropy. That being said, the changes in the thermodynamic parameters of pair association at large dilution can serve as indicators for the type of phase diagram appearing at higher IL concentrations.

**Table 1:** Thermodynamics of the Cation–Anion Association Process of the UCST and LCST ILs at 283 K and 353 K^a^

	UCST: [Hbet][Tf_2_N]/water			LCST: [P_4444_][TMBS]/water		
*T* [K]	Δ*G*	Δ*H*	*T*Δ*S*	Δ*G*	Δ*H*	*T*Δ*S*
283	–4.3 ± 0.5	–5.5 ± 0.7	–1.2 ± 1.2	–6.7 ± 0.2	–1.7 ± 0.7	+5.0 ± 0.9
353	–5.3 ± 0.4	–14.3 ± 0.9	–9.0 ± 1.3	–9.0 ± 0.1	–2.8 ± 0.7	+6.2 ± 0.8

a Estimated changes in Gibbs free energy, enthalpy, and entropy are presented for an aqueous solution containing 1 cation and 1 anion at very large dilution (see Computational Details Section in the SI for more information). All values are in kJ/mol.

Aiming to explain the favorable enthalpy changes shown in Table 1, we calculate the average number of hydrogen bonds between water molecules and ions in the bound and unbound states (Table S1). As expected, larger numbers are found in the unbound state and at a low temperature. Yet, the decrease in the number of hydrogen bonds of the unbound state upon heating is small (around 0.4) and exhibits similar values for UCST and LCST ILs. This indicates that the argument of a loss of IL-water hydrogen bond(s) at high temperatures cannot be used here to explain LCST behavior where liquid–liquid phase separation is observed upon heating.

Given the construction of the bound and unbound states, where the degrees of freedom of the ions are mainly rotational and vibrational, the positive change in entropy upon association of the LCST IL is likely to arise from the reorganization of surrounding water molecules. What is the origin of this favorable entropy change of water? Binding of hydrophobic particles in aqueous solution means elimination of hydrophobic surface area and, thereby, reduction in the number of interfacial water molecules. Due to thermal excitations, water molecules in bulk do not attain the maximum energy of attraction with their neighbors. A manifestation of this is the constant rearrangements of their hydrogen bond network.^48–50^ Although disfavored by enthalpy, these reorientations of the water molecules are favored by entropy. As a matter of fact, these reorientations also occur for water molecules next to hydrophobic solutes; however, with dynamics that are slower compared to those for water molecules in the bulk.^47,51–56^ This retarded reorientation is argued to originate from the hydrophobe’s excluded volume at the transition state of a hydrogen bond exchange.^57^ In Figure 4 we plot the autocorrelation function of hydrogen bonds between water molecules within a 0.5 nm solvation shell around UCST and LCST ions. The plots indicate that water molecules surrounding the LCST cation (tetrabutylphosphonium) exhibit significantly slower rearrangements of hydrogen bonds compared with water around the UCST cation, and this slower relaxation is larger at the lower temperature. Hydrogen bond lifetimes surrounding the anions display a small difference between LCST and UCST ILs, where at 353 K, this difference hardly exists. In all cases, the relaxations of the correlation functions around the ions are slower than those in bulk. In addition to orientations, the magnitude of translations is also smaller for water molecules next to hydrophobes compared with that for bulk water.^58–60^ In Figure 5 we show the mean squared displacement of the water molecules around the cations and anions of the ILs, covering short time intervals in which the maximum displacements are smaller than 0.55 nm. Clearly, the diffusion of waters next to the LCST cation is slower than that of waters next to the UCST cation. At 283 K, the value of the diffusion coefficient of water is 9.94·^–10^, 1.21·^–9^, and 1.51·^–9^ m^2^/s around the LCST cation, around the UCST cation, and for bulk, respectively. This ranking of water diffusion is also observed for water molecules around the anions, even though the differences are smaller. Smaller differences are also observed at the high temperature, and in all cases, bulk water displays the fastest diffusion. Thus, in an LCST behavior, upon ions’ association (representing the biphasic state), the number of water molecules that are “restricted” (i.e., with retarded orientational and diffusional dynamics) is reduced compared with the dissociated state (representing the monophasic state). In other words, some of the “restricted” water molecules surrounding the ions in the dissociated state are liberated into bulk water when the ions associate. This explains the positive change in entropy found in Table 1 at both temperatures, where the effective interactions (Δ*G* values or the PMFs in Figure 3) are stronger at the higher temperature. An increase in the strength of interactions with temperature is an anomalous behavior in physical systems because an increase in temperature augments random thermal motions, which, in turn, reduce directional attraction between particles. Nonetheless, hydrophobic interactions do exhibit this behavior, which has been coined with the term *inverse temperature phenomenology*.^61^ For this reason, interactions that are strengthened with temperature are attributed to hydrophobic interactions. Retarded dynamics are also observed for water molecules next to UCST ions. However, their magnitudes are smaller, and therefore, a smaller degree of strengthening of the effective cation–anion interactions is observed upon association. At 353 K, the magnitude of the increase in hydrophobic interaction is approximately compensated by the decrease of the effective interaction due to a rise in random thermal motion, such that the PMF is not significantly different than that at 283 K.

**Figure 4:**
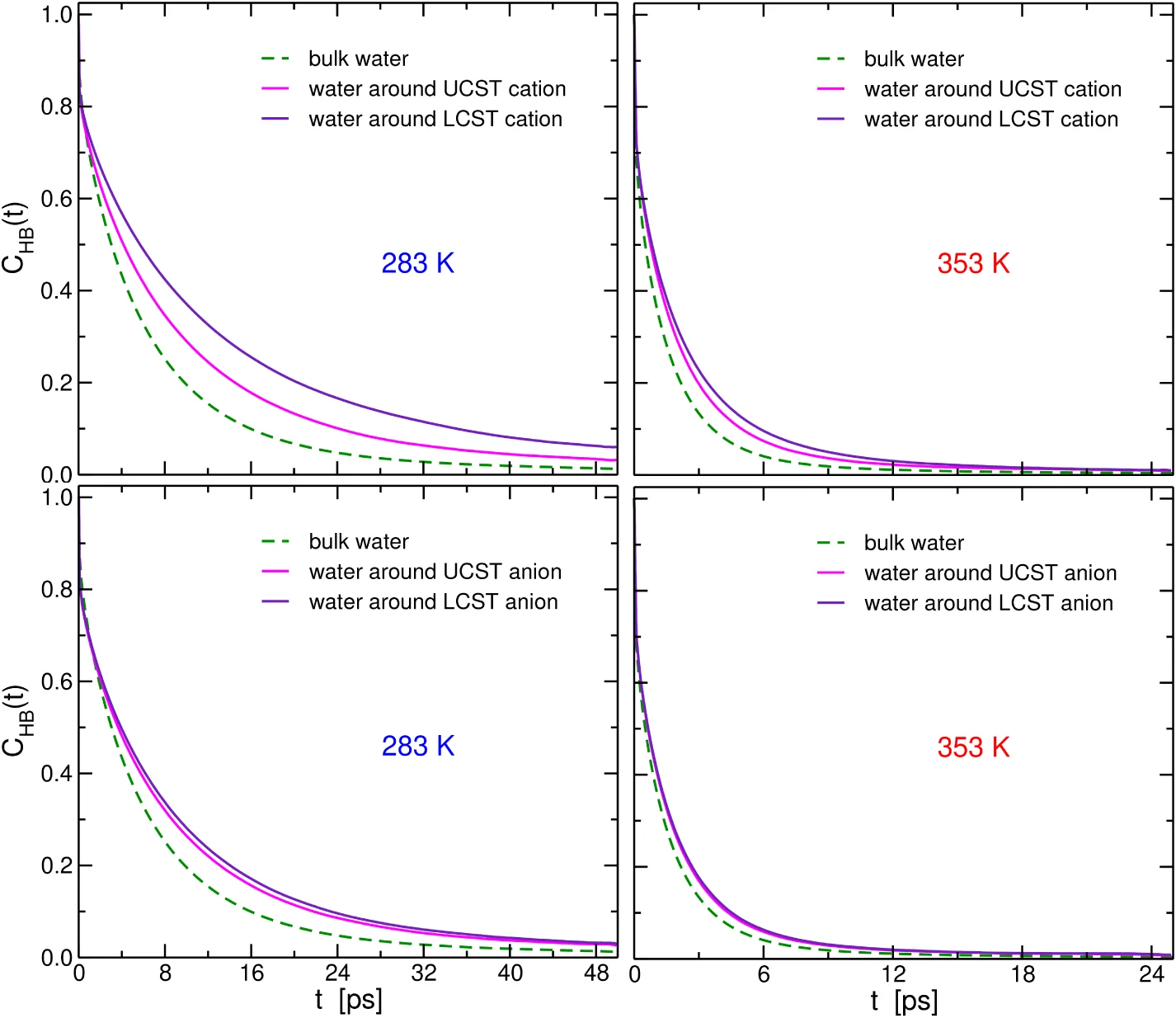
Hydrogen bonds autocorrelation function, *C*_HB_(*t*), defined in eq S1, as a function of time of water molecules around the cation (top panel) and anion (lower panel) of UCST and LCST ILs at temperatures of 283 and 353 K. The corresponding plots of bulk water molecules are shown as references.

**Figure 5:**
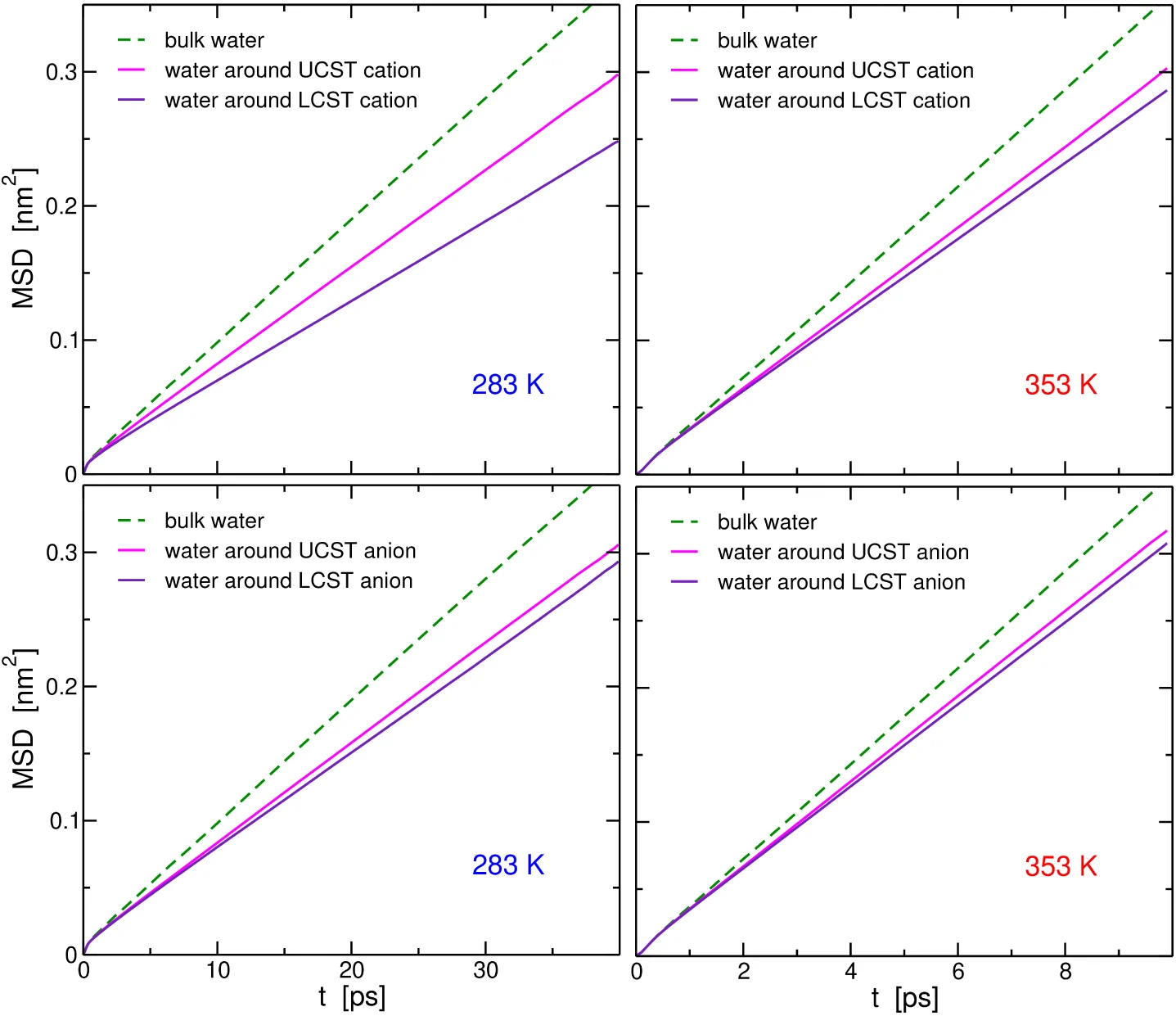
Mean squared displacements as a function of time of water molecules around the cation (top panel) and anion (lower panel) of UCST and LCST ILs at temperatures of 283 and 353 K. The corresponding plots of bulk water molecules are shown as references.

## Conclusions

We thereby conclude that the solvent-induced positive entropy change is the dominant driving force transforming a monophasic homogeneous state to a liquid–liquid phase-separated state upon a temperature increase in LCST IL/water mixtures. As mentioned above, UCST ions do exhibit some degree of hydrophobic character, presumably aiding in the separation of two liquid phases at low temperatures. Nonetheless, the extent of their hydrophobicity is not sufficient to overcompensate the penalty of the demixing entropy with an increase in temperature, and as a consequence, the mixed state is favored. Obviously, the conclusions drawn in this paper are pertinent to IL-water mixtures where the ternary systems involve extensive ion–ion and ion–water interactions. Other materials, such as those based on poly­(N-isopropylacrylamide) (PNIPAM), which contain neutral components, can also display LCST behavior.^62^ Given the similarity of the phase diagrams exhibited by these two different types of materials, we cautiously conjecture that the underlying mechanism of the liquid–liquid phase separation upon temperature increase is similar.

## Supplementary Material



## Notes

Details of the simulations’ techniques and analyses: RDFs between the IL ions for systems containing 2-IL units dissolved in 10,000 water molecules; RDFs and PMFs between a cation and an anion for systems containing only 1-IL unit dissolved in 10,000 water molecules; analyses of hydrogen bonds between water and UCST, or LCST ILs (PDF)

## References

[1] Hyman A. A., Weber C. A., Jülicher F. (2014). Liquid-Liquid
Phase Separation in
Biology. Annu. Rev. Cell Dev. Biol..

[2] Deguchi Y., Nakamura N., Ohno H. (2020). Thermoresponsive
ionic liquid/water mixtures for separation and purification technologies. Sep. Purif. Technol..

[3] Kawano M., Sadakane K., Iwase H., Matsugami M., Marekha B. A., Idrissi A., Takamuku T. (2021). Assessment of the UCST-type
liquid-liquid phase separation mechanism of imidazolium-based ionic
liquid, [C8mim]­[TFSI], and 1,4-dioxane by SANS, NMR, IR, and MD simulations. Phys. Chem. Chem. Phys..

[4] Fujii Y., Kawamura A., Morimoto N., Miyata T. (2025). Temperature-Responsive
Zwitterionic Polymers That Undergo UCST-Type Liquid-Liquid Phase Separation
under Physiological Ionic Strength. Langmuir.

[5] Takahashi T., Akiya K., Niizeki T., Matsumoto M., Hoshina T. (2022). Tunable thermoresponsive UCST-type
alkylimidazolium ionic liquids as a draw solution in the forward osmosis
process. Colloids Surf., A.

[6] Kuroyanagi S., Shimada N., Fujii S., Furuta T., Harada A., Sakurai K., Maruyama A. (2019). Highly Ordered Polypeptide with UCST
Phase Separation Behavior. J. Am. Chem. Soc..

[7] Chen L., Lin C., Zhao C., Duan X., Zhao T., Liu M. (2023). Ionic Liquids: An Ideal
Solvent for Tuning the UCST Phase Behavior of Polymer Gels. J. Phys. Chem. B.

[8] Oh S.-H., Choi S.-H. (2024). Upper Critical Solution Temperature
(UCST) Behavior of Polyguanidinium in Aqueous Media. Macromolecules.

[9] Dong S., Heyda J., Yuan J., Schalley C. A. (2016). Lower critical
solution temperature (LCST) phase behaviour of an ionic liquid and
its control by supramolecular host–guest interactions. Chem. Commun..

[10] Zhang Y., Lu F., Yu Y., Su L., Gao Y., Zheng L., Gao X. (2022). Amphiphile regulated ionic-liquid-based aqueous biphasic systems
with tunable LCST and extraction behavior. Colloids
Surf., A.

[11] Hirschfelder J., Stevenson D., Eyring H. (1937). A Theory of Liquid Structure. J. Chem. Phys..

[12] Yuan Y., Raheja K., Milbrandt N. B., Beilharz S., Tene S., Oshabaheebwa S., Gurkan U. A., Samia A. C. S., Karayilan M. (2023). Thermoresponsive
polymers with LCST transition: synthesis, characterization, and their
impact on biomedical frontiers. RSC Appl. Polym..

[13] Paricaud P., Galindo A., Jackson G. (2003). Understanding
liquid-liquid immiscibility and LCST behaviour in polymer solutions
with a Wertheim TPTl description. Mol. Phys..

[14] Zhao Y., Tian L., Pei Y., Wang H., Wang J. (2018). Effect of Anionic Structure on the
LCST Phase Behavior of Phosphonium Ionic Liquids in Water. Ind. Eng. Chem. Res..

[15] Zhao Y., Wang H., Pei Y., Liu Z., Wang J. (2016). Understanding the mechanism of LCST phase separation of mixed ionic
liquids in water by MD simulations. Phys. Chem.
Chem. Phys..

[16] Choi E., Yethiraj A. (2015). Entropic Mechanism
for the Lower Critical Solution Temperature of Poly­(ethylene oxide)
in a Room Temperature Ionic Liquid. ACS Macro
Lett..

[17] Billinge I. H., Barbosa G. D., Tao S., Terban M. W., Turner C. H., Billinge S. J., Yip N. Y. (2025). A structural underpinning of the
lower critical solution temperature (LCST) behavior behind temperature-switchable
liquids. Matter.

[18] Kang H., Suich D. E., Davies J. F., Wilson A. D., Urban J. J., Kostecki R. (2019). Molecular insight into the lower
critical solution temperature transition of aqueous alkyl phosphonium
benzene sulfonates. Commun. Chem..

[19] Forero-Martinez N. C., Cortes-Huerto R., Benedetto A., Ballone P. (2022). Thermoresponsive ionic liquid/water mixtures: From
Nanostructuring to phase separation. Molecules.

[20] Mochizuki K., Sumi T., Koga K. (2016). Liquid–liquid
phase separation of N-isopropylpropionamide aqueous solutions above
the lower critical solution temperature. Sci.
Rep..

[21] Ye Z., Sun S., Wu P. (2020). Distinct Cation-Anion
Interactions in the UCST and LCST Behavior of Polyelectrolyte Complex
Aqueous Solutions. ACS Macro Lett..

[22] Sanadhya S., Tucker Z. D., Gulotty E. M., Boggess W., Ashfeld B. L., Moghaddam S. (2022). Thermodynamic
descriptors of sensible heat driven liquid-liquid phase separation. J. Mol. Liq..

[23] Tamate R., Ueki T. (2023). Adaptive Ion-Gel: Stimuli-Responsive, and Self-Healing Ion Gels. Chem. Rec..

[24] Pappa G. D., Voutsas E. C., Tassios D. P. (2001). Liquid-Liquid Phase Equilibrium in
Polymer-Solvent Systems: Correlation and Prediction of the Polymer
Molecular Weight and the Pressure Effect. Ind.
Eng. Chem. Res..

[25] Frank T. C., Donate F. A., Merenov A. S., Von Wald G. A., Alstad B. J., Green C. W., Thyne T. C. (2007). Separation of Glycol Ethers and Similar
LCST-Type Hydrogen-Bonding Organics from Aqueous Solution Using Distillation
or Liquid-Liquid Extraction. Ind. Eng. Chem.
Res..

[26] Batista M.
L. S., Tomé L. I. N., Neves C. M. S. S., Rocha E. M., Gomes J. R. B., Coutinho J. A. P. (2012). The Origin of the LCST on the Liquid-Liquid
Equilibrium of Thiophene with Ionic Liquids. J. Phys. Chem. B.

[27] Gobbo D., Ballone P., Garabato B. (2020). Coarse-Grained Model of Entropy-Driven
Demixing. J. Phys. Chem. B.

[28] Bahramian A. (2024). Unlocking the Secrets of Liquid-Liquid
Interfaces and Phase Equilibria: Exploring the Interplay of Critical
Composition, Interfacial Tension, and Mutual Solubility. Langmuir.

[29] Mahfouz A., Kocher J. D., Haddad A. Z., Menon A. K. (2026). Elucidating
the Microscale Behavior and Phase Separation Kinetics of Thermally
Responsive Ionic Liquid-Water Mixtures. ACS
Appl. Mater. Interfaces.

[30] Mohammed H. O., Zangi R. (2026). MD Simulations of UCST Liquid-Liquid Phase Separations of [Hbet]­[Tf_2_N]/Water Mixtures. J. Mol. Liq..

[31] Mohammed H. O., de Cózar A., Zangi R. (2025). Modeling and Elucidating the Behavior of a Thermoresponsive LCST
Ionic-Liquid. J. Chem. Inf. Model..

[32] Zhong Y., Feng X., Chen W., Wang X., Huang K.-W., Gnanou Y., Lai Z. (2016). Using UCST
Ionic Liquid as a Draw Solute in Forward Osmosis to Treat High-Salinity
Water. Environ. Sci. Technol..

[33] Zeweldi H. G., Bendoy A. P., Park M. J., Shon H. K., Kim H.-S., Johnson E. M., Kim H., Lee S.-P., Chung W.-J., Nisola G. M. (2020). Tetrabutylammonium
2, 4, 6-trimethylbenzenesulfonate as an effective and regenerable
thermo-responsive ionic liquid drawing agent in forward osmosis for
seawater desalination. Desalination.

[34] Kohno Y., Saita S., Men Y., Yuan J., Ohno H. (2015). Thermoresponsive polyelectrolytes
derived from ionic liquids. Polym. Chem..

[35] Kohno Y., Nakamura N., Ohno H. (2012). Selective
transport of water-soluble proteins from aqueous to ionic liquid phase
via a temperature-sensitive phase change of these mixtures. Aust. J. Chem..

[36] Bühl M., Chaumont A., Schurhammer R., Wipff G. (2005). Ab Initio Molecular
Dynamics of Liquid 1,3-Dimethylimidazolium Chloride. J. Phys. Chem. B.

[37] Cremer T., Kolbeck C., Lovelock K. R. J., Paape N., Wölfel R., Schulz P. S., Wasserscheid P., Weber H., Thar J., Kirchner B. (2010). Towards a Molecular Understanding of Cation-Anion
Interactions-Probing the Electronic Structure of Imidazolium Ionic
Liquids by NMR Spectroscopy, X-ray Photoelectron Spectroscopy and
Theoretical Calculations. Chem.- Eur. J..

[38] Schmidt J., Krekeler C., Dommert F., Zhao Y., Berger R., Site L. D., Holm C. (2010). Ionic Charge
Reduction and Atomic Partial Charges from First-Principles Calculations
of 1,3-Dimethylimidazolium Chloride. J. Phys.
Chem. B.

[39] Wendler K., Zahn S., Dommert F., Berger R., Holm C., Kirchner B., Delle Site L. (2011). Locality and
Fluctuations: Trends in Imidazolium-Based Ionic Liquids and Beyond. J. Chem. Theory Comput..

[40] Hollóczki O., Malberg F., Welton T., Kirchner B. (2014). On the origin of ionicity
in ionic liquids. Ion pairing versus charge transfer. Phys. Chem. Chem. Phys.

[41] Petrov D., Perthold J. W., Oostenbrink C., de Groot B. L., Gapsys V. (2024). Guidelines
for Free-Energy Calculations Involving Charge Changes. J. Chem. Theory Comput..

[42] Lin Y., Fichou Y., Longhini A. P., Llanes L. C., Yin P., Bazan G. C., Kosik K. S., Han S. (2021). Liquid-Liquid Phase
Separation of Tau Driven by Hydrophobic Interaction Facilitates Fibrillization
of Tau. J. Mol. Biol..

[43] Nockemann P., Thijs B., Pittois S., Thoen J., Glorieux C., Van Hecke K., Van Meervelt L., Kirchner B., Binnemans K. (2006). Task-Specific Ionic Liquid for Solubilizing
Metal Oxides. J. Phys. Chem. B.

[44] Kohno Y., Arai H., Saita S., Ohno H. (2011). Material design of ionic liquids to show temperature-sensitive LCST-type
phase transition after mixing with water. Aust.
J. Chem..

[45] Frank H. S., Evans M. W. (1945). Free Volume and Entropy in Condensed Systems III. Entropy
in Binary Liquid Mixtures; Partial Molal Entropy in Dilute Solutions;
Structure and Thermodynamics in Aqueous Electrolytes. J. Chem. Phys..

[46] Lum K., Chandler D., Weeks J. D. (1999). Hydrophobicity at small and large
length scales. J. Phys. Chem. B.

[47] Zangi R. (2011). Driving Force for Hydrophobic Interaction
at Different Length-Scales. J. Phys. Chem. B.

[48] Sciortino F., Geiger A., Stanley H. E. (1991). Effect
of defects on molecular mobility in liquid water. Nature.

[49] Laage D., Hynes J. T. (2008). On the molecular
mechanism of water reorientation. J. Phys. Chem.
B.

[50] Tay K. A., Bresme F. (2009). Kinetics of hydrogen-bond
rearrangements in bulk water. Phys. Chem. Chem.
Phys..

[51] Geiger A., Rahman A., Stillinger F. H. (1979). Molecular
dynamics study of the hydration of Lennard-Jones solutes. J. Chem. Phys..

[52] Rossky P. J., Karplus M. (1979). Solvation. a molecular dynamics study of a dipeptide
in water. J. Am. Chem. Soc..

[53] Carlstroem G., Halle B. (1988). Water dynamics in microemulsion
droplets. a nuclear spin relaxation study. Langmuir.

[54] Ishihara Y., Okouchi S., Uedaira H. (1997). Dynamics of
hydration of alcohols and diols in aqueous solutions. J. Chem. Soc., Faraday Trans..

[55] Xu H., Berne B. J. (2001). Hydrogen-Bond Kinetics
in the Solvation Shell of a Polypeptide. J.
Phys. Chem. B.

[56] Rezus Y. L. A., Bakker H. J. (2007). Observation of immobilized water molecules around hydrophobic
groups. Phys. Rev. Lett..

[57] Laage D., Stirnemann G., Hynes J. T. (2009). Why Water Reorientation
Slows without Iceberg Formation around Hydrophobic Solutes. J. Phys. Chem. B.

[58] Lee S. H., Rossky P. J. (1994). A comparison of the structure and dynamics of liquid
water at hydrophobic and hydrophilic surfaces – a molecular
dynamics simulation study. J. Chem. Phys..

[59] Haselmeier R., Holz M., Marbach W., Weingaertner H. (1995). Water Dynamics near a Dissolved Noble Gas. First Direct
Experimental Evidence for a Retardation Effect. J. Phys. Chem..

[60] Hua L., Huang X., Zhou R., Berne B. J. (2006). Dynamics of Water
Confined in the Interdomain Region of a Multidomain Protein. J. Phys. Chem. B.

[61] Pratt L. R., Chaudhari M. I., Rempe S. B. (2016). Statistical Analyses of Hydrophobic
Interactions: A Mini-Review. J. Phys. Chem.
B.

[62] Li Y., Luo J., Xie G., Zhu D., Zhao C., Zhang X., Liu M., Wu Y., Guo Y., Yu W. (2025). Recent Progress on Regulating the
LCST of PNIPAM-Based Thermochromic Materials. ACS Appl. Polym. Mater..

