# Supplementary material for: Driving Forces of UCST and LCST Liquid–Liquid Phase Separations
Source: ACS Omega. 2026 Aug 10;11(33):50147–55. doi: 10.1021/acsomega.6c05528 (PMC13520039; doi:10.1021/acsomega.6c05528)
Supplement: Supplementary file 1 [file ao6c05528_si_001.pdf]

Supporting Information: Driving Forces of UCST and LCST Liquid-Liquid Phase Separations

Hussen O. Mohammed[1,2] and Ronen Zangi*[1,2,3]

[1] *Donostia International Physics Center (DIPC), 20018 Donostia-San Sebastián, Spain*

[2] *Department of Organic Chemistry I, University of the Basque Country UPV/EHU, 20018 Donostia-San Sebastián, Spain*

[3] *IKERBASQUE, Basque Foundation for Science, 48009 Bilbao, Spain*

August 2, 2026

*r.zangi@ikerbasque.org ; ronen.zangi@ehu.eus

Computational Details

Two types of ionic-liquids (see Fig. 1), protonated trimethylglycine bis(trifluoromethylsulfonyl)amide ([Hbet][Tf_2N]) and tetrabutylphosphonium 2,4,6-trimethylbenzene-sulfonate ([P_{4444}][TMBS]) that when mixed with water display, respectively, a UCST and a LCST liquid-liquid phase separation were modeled by molecular dynamics simulations. The systems modeled include either one, or two, ionic-liquid (IL) unit(s) in a box filled with 10^4 water molecules. Depending on the systems, the resulting cubic simulation boxes ranged from 6.6 to 6.8 nm, yielding IL concentrations of around 0.0055 M and 0.011 M for the 1-IL and 2-IL systems, respectively.

To describe these polyatomic ions, the partial charges of the atoms were determined by the restrained electrostatic potential (RESP) fitting procedure[1–3]. In all cases, we found it necessary to down-scale the charges by a factor of 0.95 in order to obtain agreement with the experimental phase diagrams. Parameters describing atom types, bond vibrations, angle bendings, and dihedral torsions were based on the OPLSAA and AMBER force fields[1,4–6]. Vibrations of covalent bonds are described by harmonic potentials, except for bonds involving hydrogen atoms, in which case the bonds were constrained to their equilibrium distances using the LINCS[7] method. Detailed specifications of all partial charges, non-bonded and bonded parameters of the UCST and LCST ions are provided in our previous works[2,3]. Water molecules were described by the TIP4P/2005 model[8] with the two covalent bonds and angle constrained using the SETTLE[9] algorithm.

All simulations were performed by the GROMACS software version 2025.2[10]. Newton's equations of motion were integrated by leapfrog algorithm with a time step of 2 fs. Periodic boundary conditions were applied along all three Cartesian axes. Constant temperature was maintained by coupling the system to a thermal bath using the velocity rescaling algorithm[11] with a 1.0 ps relaxation time. Constant pressure was achieved by applying the C-rescale barostat[12] with relaxation time of 5.0 ps and compressibility of 10^{-5} ps. Electrostatic interactions were calculated using the particle-mesh Ewald (PME) method[13,14], setting the real-space cutoff to 1.0 nm, and the reciprocal-space grid spacing to 0.12 nm (quadratic interpolation). Lennard-Jones potentials were

calculated up to a cutoff of $1.0\ nm$, where longer range interactions were accounted for by including long-range dispersion corrections for energy and pressure.

The systems were prepared by placing the ions and water molecules randomly in the simulation box utilizing PACKMOL version 20.010[15], after which the system was energy minimized by the steepest descent approach. Then, the systems were equilibrated for $500\ ns$ at a pressure of $1\ bar$ and temperatures of $283\ K$ and $353\ K$. To augment the statistics in the data collection step, we generated 30 independent replica by extracting 30 configurations from the equilibration step. Each replica was then run for $100\ ns$ for each IL at each temperature. Moreover, in order to simulate the associated (bound) and dissociated (unbound) states of the UCST 1-IL system, the distance between the N atom of $[Hbet]^+$ and the N atom of $[Tf_2N]^-$ was fixed to $0.65\ nm$ and $3.00\ nm$, respectively. For the LCST, the distance between the P atom of $[P_{4444}]^+$ and the S atom of $[TMBS]^-$ was fixed to $0.55\ nm$ and $3.00\ nm$ for the associate and dissociated states. Technically, to maintain these distances constant during simulations we employed the pull-code in Gromacs. The change in enthalpy between the associate (bound) and dissociated (unbound) states, ΔH, is approximated by the corresponding change in energy. This change in energy is calculated by subtracting a large number from a similar large number. Hence, to compute ΔH with an acceptable degree of accuracy, also here, we dedicated additional 30 simulations (replica) for $100\ ns$ each, for each IL at each temperature, where the cation-anion distance was fixed at the value representing these two state. For each of these independent replica runs, the error in energy was estimated by the block averaging method[16]. Then, the average of these estimated errors for each of the $n = 30$ replica was divided by $\sqrt{n}$ to obtain an estimation for the error considering all independent simulations. To estimate the error of ΔG, which is obtained from the radial distribution function $g(r)$ via the relation $w(r) = -RT \ln g(r)$, we divided the 30 replica into two sets of 15. Then, for each of these 15-replica set we calculated the value of ΔG, as well as, for the entire 30-replica. The largest distance of the value obtained from the full set to those obtained from the half-sets was taken as an estimation of the error.

Two water molecules were considered to be hydrogen bonded if the distance between the oxygen

atom of the donor and acceptor was smaller than $0.35\ nm$, and the hydrogen–acceptor–donor angle was smaller than $30°$[17]. Error estimation in calculating the average number of hydrogen bonds followed the procedure mentioned above for the calculation of the energy. The dynamics of the hydrogen bond lifetime between water molecules around the cation or anion was computed by the auto-correlation function[17,18],

$$C_{HB}(t) = \frac{\langle h(0)h(t)\rangle}{\langle h\rangle} \qquad , \tag{S1}$$

where the average includes all pairs of water molecules inside the 'first' solvation shell of the ions. This was determined by a cut-off distance of $0.50\ nm$ between the oxygen atom of water and any atom of the cation or anion. The value of $h(t)$ equals 1 if a particular pair of water molecules, that was hydrogen-bonded at time 0, is hydrogen bonded at time t (allowing broken bonds at intermediate times), and otherwise 0. The mean squared displacement of water molecules around the cation and anion was calculated in a similar way. Hence, the displacements were recorded only for water molecules that at time 0 were located inside the $0.50\ nm$ solvation shell around (any atom of) the cation or anion. To calculate these time-dependent properties we performed dedicated simulations, in the dissociated state, in which the coordinates were saved every $0.1\ ps$ in a $2\ ns$ trajectory. To improve statistics, we generated 6 independent replica for each IL at each temperature.

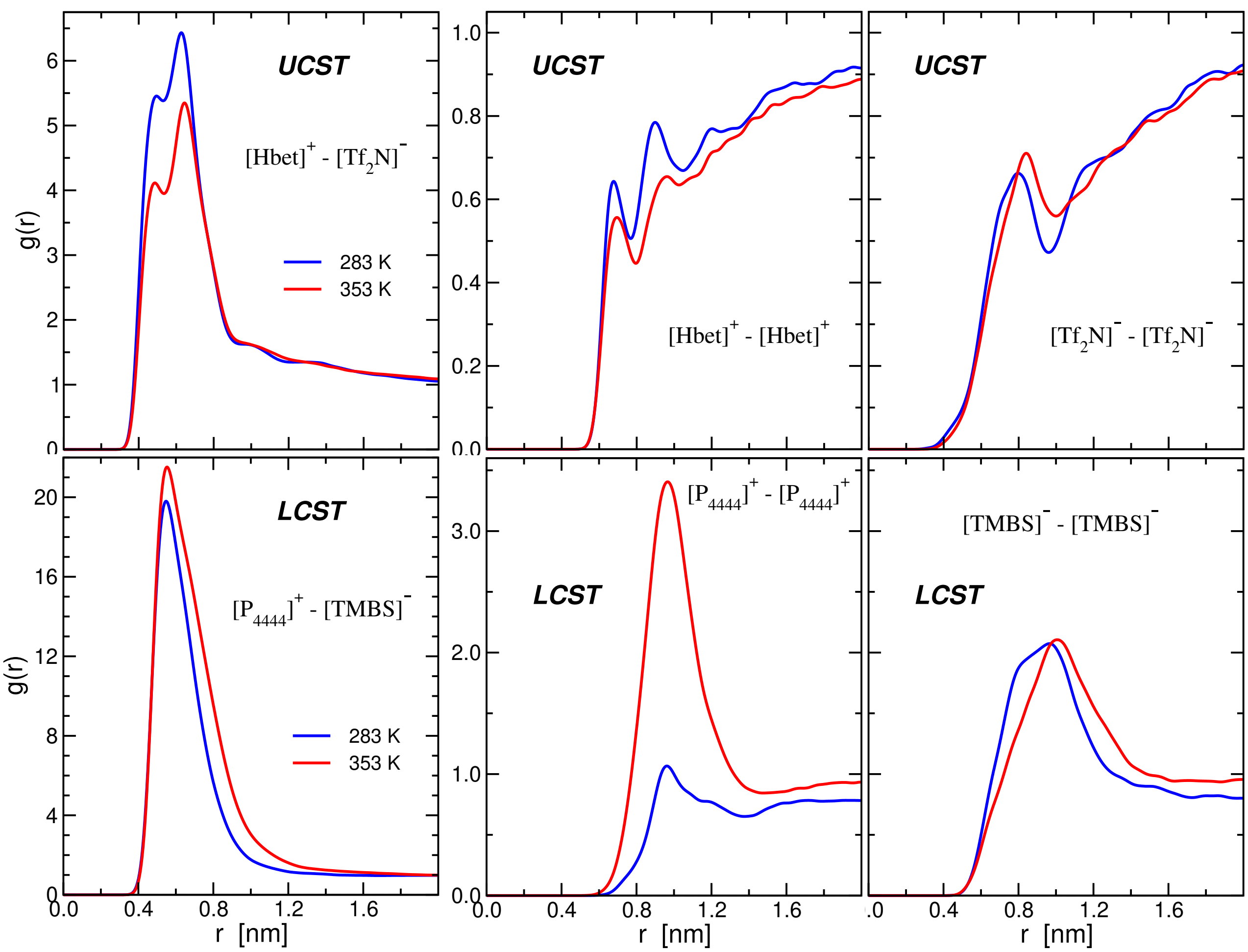

Figure S1: Radial distribution functions between the IL's ions at low (283 K) and high (353 K) temperatures for UCST ([Hbet][Tf_2N] mixed with water; upper panel) and LCST ([P_{4444}][TMBS] mixed with water; lower panel) types of liquid-liquid phase separation. The results are obtained from a system containing only 2 IL units dissolved in 10,000 water molecules. In these calculations, the ions are represented by their 'central' atoms; N for $[Hbet]^+$, N for $[Tf_2N]^-$, P for $[P_{4444}]^+$, and S for $[TMBS]^-$.

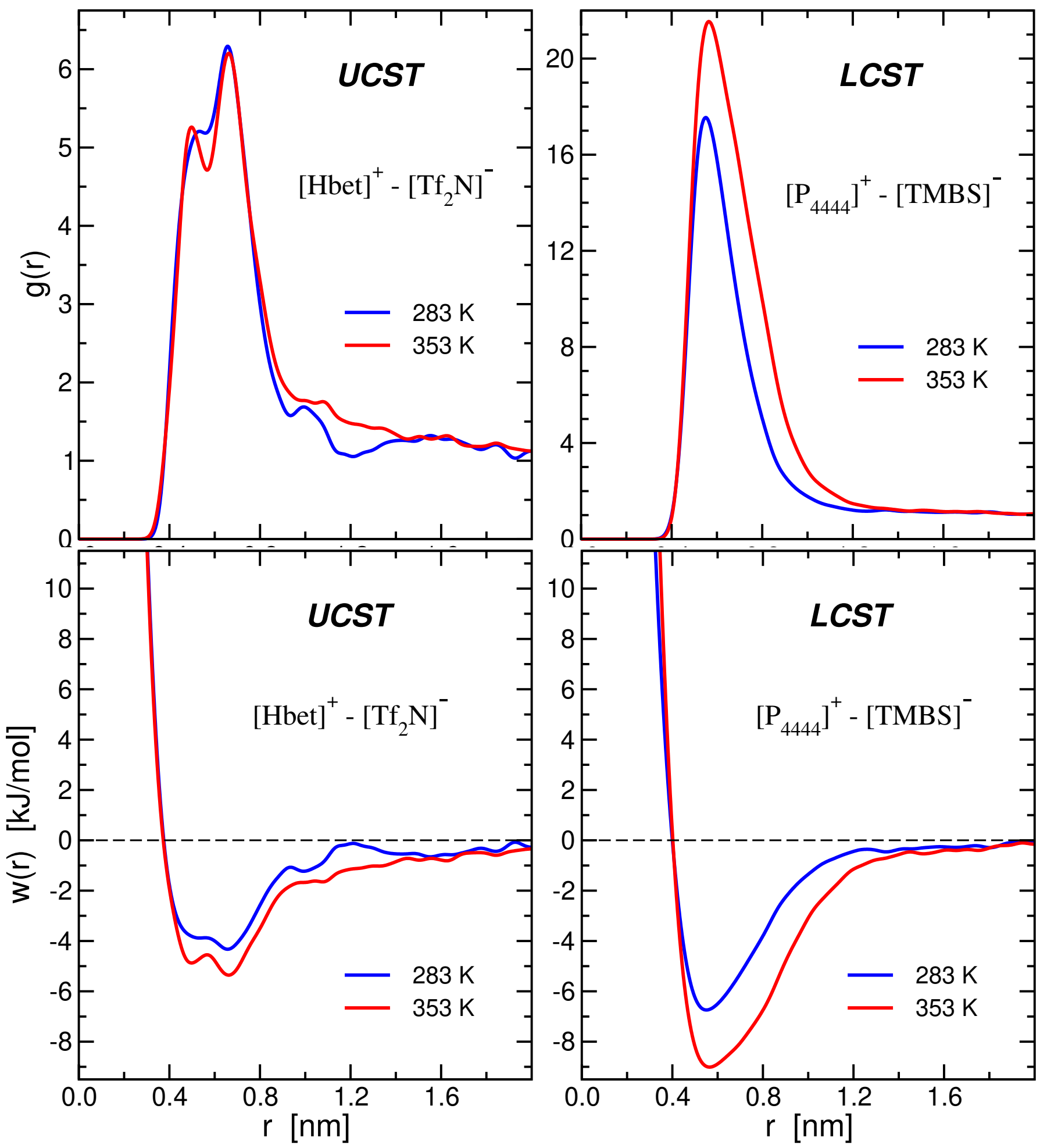

Figure S2: Radial distribution function, $g(r)$, (upper panel) and potential of mean force, $w(r)$, (lower panel) between a cation and an anion at low (283 K) and high (353 K) temperatures for UCST ([Hbet][Tf_2N] mixed with water) and LCST ([P_{4444}][TMBS] mixed with water) types of liquid-liquid phase separation. The results are obtained from a system containing only 1 IL unit dissolved in 10,000 water molecules. The representation of the ions is the same as in Fig. S1.

Table S1: Average number of hydrogen bonds between water and UCST, or LCST, cation and anion in the associated ($r = 0.65$ and $0.55\ nm$ for UCST and LCST ions, respectively) and dissociated ($r = 3.0\ nm$) states at $283\ K$ and $353\ K$. In all cases, the error estimate is smaller than 0.07.

	**UCST**				**LCST**			
	**[Hbet]$^+$ – water**		**[Tf_2N]$^-$ – water**		**[P_{4444}] $^+$ – water**		**[TMBS]$^-$ – water**	
T [K]	bound	unbound	bound	unbound	bound	unbound	bound	unbound
283	0.97	0.98	5.50	5.87	0.00	0.00	5.36	5.93
353	0.96	0.99	5.13	5.50	0.00	0.00	4.91	5.55

References

[1] Zhou, G.; Liu, X.; Zhang, S.; Yu, G.; He, H. A Force Field for Molecular Simulation of Tetrabutylphosphonium Amino Acid Ionic Liquids, *J. Phys. Chem. B* **2007**, *111*, 7078–7084.

[2] Mohammed, H. O.; de Cózar, A.; Zangi, R. Modeling and Elucidating the Behavior of a Thermoresponsive LCST Ionic-Liquid, *J. Chem. Inf. Model.* **2025**, *65*, 785–797.

[3] Mohammed, H. O.; Zangi, R. MD Simulations of UCST Liquid-Liquid Phase Separations of [Hbet][Tf_2N]/Water Mixtures, *J. Mol. Liq.* **2026**, *446*, 129314.

[4] Jorgensen, W. L.; Severance, D. L. Aromatic-Aromatic Interactions: Free Energy Profiles for the Benzene Dimer in Water, Chloroform and Liquid Benzene, *J. Am. Chem. Soc.* **1990**, *112*, 4768–4774.

[5] Jorgensen, W. L.; Maxwell, D. S.; Tirado-Rives, J. Development and Testing of the OPLS All-Atom Force Field on Conformational Energetics and Properties of Organic Liquids, *J. Am. Chem. Soc.* **1996**, *118*, 11225–11236.

[6] Price, M. L. P.; Ostrovsky, D.; Jorgensen, W. L. Gas-Phase and Liquid-State Properties of Esters, Nitriles, and Nitro Compounds with the OPLS-AA Force Field, *J. Comp. Chem.* **2001**, *22*, 1340–1352.

[7] Hess, B.; Bekker, H.; Berendsen, H. J. C.; Fraaije, J. G. E. M. LINCS: A Linear Constraint Solver for Molecular Simulations, *J. Comp. Chem.* **1997**, *18*, 1463–1472.

[8] Abascal, J. L. F.; Vega, C. A general purpose model for the condensed phases of water: TIP4P/2005, *J. Chem. Phys.* **2005**, *123*, 234505.

[9] Miyamoto, S.; Kollman, P. A. SETTLE: An Analytical Version of the SHAKE and RATTLE Algorithms for Rigid Water Models, *J. Comp. Chem.* **1992**, *13*, 952–962.

[10] Abraham, M. J.; Murtola, T.; Schulz, R.; Páll, S.; Smith, J. C.; Hess, B.; Lindahl, E. GROMACS: High performance molecular simulations through multi-level parallelism from laptops to supercomputers, *SoftwareX* **2015**, *1*, 19–25.

[11] Bussi, G.; Donadio, D.; Parrinello, M. Canonical Sampling through Velocity Rescaling, *J. Chem. Phys.* **2007**, *126*, 014101.

[12] Bernetti, M.; Bussi, G. Pressure control using stochastic cell rescaling, *J. Chem. Phys.* **2020**, *153*, 114107.

[13] Darden, T.; York, D.; Pedersen, L. Particle Mesh Ewald: An N-log(N) Method for Ewald Sums in Large Systems, *J. Chem. Phys.* **1993**, *98*, 10089–10092.

[14] Essmann, U.; Perera, L.; Berkowitz, M. L.; Darden, T.; Lee, H.; Pedersen, L. G. A Smooth Particle Mesh Ewald Method, *J. Chem. Phys.* **1995**, *103*, 8577–8593.

[15] Martínez, L.; Andrade, R.; Birgin, E. G.; Martínez, J. M. PACKMOL: A package for building initial configurations for molecular dynamics simulations, *J. Compu. Chem.* **2009**, *30*, 2157–2164.

[16] Flyvbjerg, H.; Petersen, H. G. Error Estimates on Averages of Correlated Data, *J. Chem. Phys.* **1989**, *91*, 461–466.

[17] Luzar, A.; Chandler, D. Effect of Environment on Hydrogen Bond Dynamics in Liquid Water, *Phys. Rev. Lett.* **1996**, *76*, 928–931.

[18] Pal, S.; Maiti, P. K.; Bagchi, B. Exploring DNA groove water dynamics through hydrogen bond lifetime and orientational relaxation, *J. Chem. Phys.* **2006**, *125*, 234903.
